# Activated Drp1-mediated mitochondrial ROS influence the gut microbiome and intestinal barrier after hemorrhagic shock

**DOI:** 10.18632/aging.102690

**Published:** 2020-01-18

**Authors:** Chenyang Duan, Lei Kuang, Xinming Xiang, Jie Zhang, Yu Zhu, Yue Wu, Qingguang Yan, Liangming Liu, Tao Li

**Affiliations:** 1 State Key Laboratory of Trauma, Burns and Combined Injury, Second Department of Research Institute of Surgery, Daping Hospital, Army Medical University, Chongqing 400042, China

**Keywords:** Drp1, mitochondrial ROS, SCFA, gut microbiome, intestinal barrier

## Abstract

A role of the mitochondrial dynamin-related protein (Drp1) on gut microbiome composition and intestinal barrier function after hemorrhagic shock has not been identified previously and thus addressed in this study. Here, we used a combination of 16S rRNA gene sequencing and mass spectrometry-based metabolomics profiling in WT and Drp1 KO mouse models to examine the functional impact of activated Drp1 on the gut microbiome as well as mitochondrial metabolic regulation after hemorrhagic shock. Our data showed that changes in mitochondrial Drp1 activity participated in the regulation of intestinal barrier function after hemorrhagic shock. Activated Drp1 significantly perturbed gut microbiome composition in the *Bacteroidetes* phylum. The abundance of short-chain fatty acid (SCFA) producing microbes, such as *Bacteroides*, *Butyricimonas* and *Odoribacter*, was markedly decreased in mice after shock, and was inversely correlated with both the distribution of the tight junction protein ZO1 and intestinal permeability. Together, these data suggest that Drp1 activation perturbs the gut microbiome community and SCFA production in a ROS-specific manner and thereby substantially disturbs tight junctions and intestinal barrier function after hemorrhagic shock. Our findings provide novel insights for targeting Drp1-mediated mitochondrial function as well as the microbiome in the treatment of intestinal barrier dysfunction after shock.

## INTRODUCTION

The intestinal barrier is composed of tight junctions between intestinal epithelial cells (IECs), which is critical to resist the invasion of harmful substances into the body [1]. In ischemic-hypoxic injury or hemorrhagic shock, the redistribution of body blood volume markedly reduces the blood flow through the intestinal mucosa to optimize the blood supply for vital organs such as the heart and brain [2]. However, another consequence of hypoxic-ischemic injury to IECs is ATP reduction and tight junction rupture, which further induce intestinal barrier dysfunction [3].

Mitochondria are the main sites for cellular physiological activities such as aerobic respiration and oxidative phosphorylation [4]. In addition to providing energy for the body, mitochondria in IECs also participate in intestinal cell proliferation and cell cycle regulation [5]. Studies have shown that dinitrophenol-induced mitochondrial dysfunction in IECs may lead to intestinal barrier dysfunction [6], suggesting that the stability of mitochondria is critical to maintaining tight junction integrity and intestinal barrier function.

Gut microbes are known as “moving organs” in the human body, which participate in nutrient metabolism and intestinal cell proliferation. There are about 10^14^ microbes in the human gastrointestinal tract, consisting of 500–1000 different species of microflora [7]. In recent years, it has been found that there is a close relationship between the gut microbiome and mitochondria in IECs [8]. On one hand, the gut microbiome can regulate mitochondrial energy metabolism and mitochondrial biosynthesis [9–11]. On the other hand, mitochondria in IECs may help to maintain intestinal barrier integrity [6] and regulate gut microbiome composition [12]. Previous studies on the mitochondria-microbiome interplay are mostly focused on the regulatory effects of microbiome metabolites on mitochondrial function, while research into mitochondrial feedback on the gut microbiome is still in its infancy.

Drp1, an important mitochondrial-related protein with GTPase activity, mediates mitochondrial fission by oligomerization [13, 14] and by inducing with its ligands Fis1, MFF and MiD49/51 [15]. Recent studies have shown that various modifications of Drp1 also participate in the regulation of mitochondrial morphology, function, metabolism and other aspects of mitochondrial mass [16], suggesting that Drp1 plays an important role in the regulation of mitochondrial quality control.

In the present study, we interrogated mitochondria in Drp1 KO mice and applied an integrated approach combining 16S rRNA gene sequencing and liquid chromatography-mass spectrometry (LC-MS) metabolomics to investigate the interaction between the gut microbiome and mitochondria in IECs after hemorrhagic shock. Our findings revealed that Drp1 activation perturbs the gut microbiome and SCFA production in a ROS-specific manner.

## RESULTS

### Mitochondrial Drp1 in intestinal epithelium is activated during hemorrhagic shock

**Figure 1:**
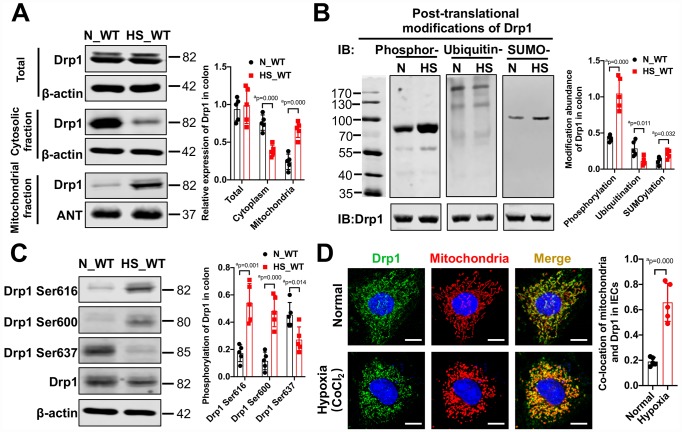
**The activation and mitochondrial translocation of Drp1 in IECs after shock or hypoxia.** (**A**) The mitochondrial translocation of Drp1 after hemorrhagic shock in colon tissues. N_WT, WT mice in normal condition; HS_WT, WT mice in hemorrhagic shock condition. (n=5 mice/ group) (**B**) Post-translational modifications of Drp1 after hemorrhagic shock detected by co-immunoprecipitation. N, N_WT group; HS, HS_WT group; Phosphor-, Phosphorylation; Ubiquitin-, ubiquitination; SUMO-, SUMOylation. (n=5 mice/ group) (**C**) The phosphorylation of Drp1 after hemorrhagic shock in colon tissues detected by Western Blotting. (n=5 mice/ group) (**D**) The co-location of Drp1 and mitochondria after CoCl_2_-induced hypoxia in IECs. (Bar, 25μm) (n=5). a represents p < 0.05 compared with N_WT group or Normal group.

To investigate the changes of mitochondrial dynamin-related protein Drp1 in intestinal epithelium after hemorrhagic shock, we examined the expression and modification of Drp1 in colon tissue from eight WT mice after 4h shock. Western Blotting results showed that there was no significant difference in total expression of Drp1 between normal (N_WT group) and post-shock colon tissue (HS_WT group) (p > 0.05). However, the expression of Drp1 in cytoplasm decreased and the expression of Drp1 in mitochondria increased significantly after shock (p < 0.05), suggesting that Drp1 mitochondrial translocation may occur after hemorrhagic shock in colon tissue (Figure 1A). In addition, we examined various types of Drp1 modification in colon tissue after shock. The PTM screening results showed that the phosphorylation of Drp1 in colon tissue markedly increased (p < 0.05), while the SUMOylation of Drp1 decreased and ubiquitination of Drp1 increased slightly (p < 0.05) (Figure 1B). The changes of other Drp1 modifications, including acetylation, malonylation and succinylaction, were not observed in colon tissue after shock (p > 0.05) (Supplementary Figure 1A).

To further explore the specific sites of Drp1 activation after hemorrhagic shock, we screened potential Drp1 phosphorylation sites in Drp1 amino acid sequence and found that phosphorylation of Drp1 serine (Ser) was significantly increased, especially at Ser616 and Ser 600 sites, after shock (p < 0.05) (Supplementary Figure 1B and 1C). At cellular level, the phosphorylation of Drp1 Ser616 and Ser600 in hypoxia-treated IECs increased by 2-3 times and phosphorylation of Drp1 Ser637 decreased by 50% (p < 0.05) (Supplementary Figure 1C), which was consistent with the Western Blotting results in colon tissue. Besides, the confocal images showed that, under normal conditions, Drp1 was diffuse and the co-localization ratio between Drp1 and mitochondria was 0.18±0.05. After hypoxia, Drp1 aggregated on mitochondria and the co-localization ratio increased to 0.62±0.15 (p < 0.05) (Figure 1D), further confirming Drp1 mitochondrial translocation occurred in intestinal epithelial cells after hypoxia. Overall, these results suggested that there existed obvious mitochondrial translocation and activity changes in intestinal epithelial Drp1 after hemorrhagic shock.

### Activated Drp1 participates in the regulation of intestinal barrier function after shock

**Figure 2:**
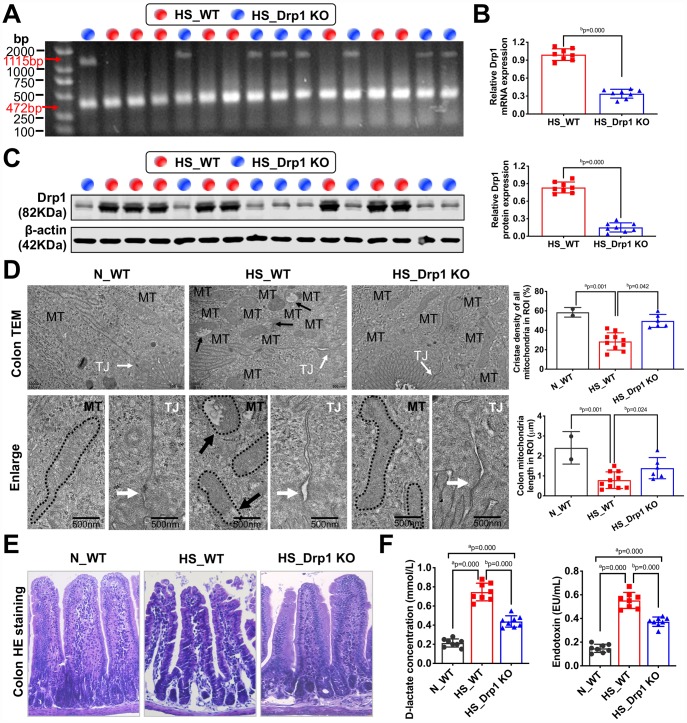
**The effects of Drp1 on intestinal barrier function after shock.** (**A**) The genotype identification results of Drp1 KO and WT mice. The genotype of Drp1 KO mice (blue dot, n=8) is Drp1 +/- (double bands at 1115 bp and 472 bp), and the genotype of WT mice (red dot, n=8) is Drp1 +/+ (single band at 472 bp). (**B**) Drp1 mRNA expression in colon tissues of HS_WT group and HS_Drp1 KO group detected by QRT-PCR (n=8 mice/ group). (**C**) Drp1 protein expression in colon tissues of HS_WT group and HS_Drp1 KO group detected by Western Blotting (n=8 mice/ group). (**D**) The mitochondrial morphology and intestinal epithelial tight junctions observed by electronic microscopy (TEM). MT, mitochondria (black arrows refer to vacuolated cristae structure); TJ, tight junctions (white arrows). The boundaries of mitochondria are depicted and the lengths as well as cristae density of all mitochondria in ROI (region of interest) are calculated by Image J software. The mitochondrial number in ROI: 2 in N_WT group, 10 in HS_WT group and 6 in HS_Drp1 KO group. (Bar, 500nm). (**E**) The intestinal villus morphology observed by immunohistochemistry (400X). (**F**) The plasma D-lactate and endotoxin concentration in each group (n=8 mice/ group). N_WT, WT mice in normal condition; HS_WT, WT mice in hemorrhagic shock condition; HS_Drp1 KO, Drp1 KO mice in hemorrhagic shock condition. a represents p < 0.05 compared with N_WT group; b represents p < 0.05 compared with HS_WT group.

To explore the effect of activated Drp1 on intestinal barrier function after shock, we established shock models in eight WT mice (HS_WT group) and eight Drp1 KO mice (HS_Drp1 KO group) to observe the differences in mitochondrial quality indexes and intestinal barrier functions between the two groups. The genotype identification results of these sixteen individuals were shown in Figure 2A. QRT-PCR and Western Blotting results showed that, compared with HS_WT group, Drp1 mRNA content in colon tissue of HS_Drp1 KO group decreased by 63% (Figure 2B), and Drp1 protein expression decreased by 70% (Figure 2C), indicating that the effect of Drp1 knockout in colon tissue was very obvious.

The electronic microscopy images of intestinal epithelium showed that, compared with N_WT group, the length of mitochondria in HS_WT group was shortened by about 60% (p < 0.05). The internal cristae structure was vacuolated (black arrow) and the tight junction of intestinal epithelium was severely damaged in HS_WT group (white arrow). However, the mitochondrial morphology, internal cristae structures and intestinal epithelial tight junctions in HS_Drp1 KO group were better than those in HS_WT group (Figure 2D), which indicated that activated Drp1 in IECs not only disturbed the structure and function of mitochondria, but also influenced the intestinal barrier function after shock. The immunohistochemistry images showed that the intestinal mucosa of HS_WT group was severely damaged, while the morphology of intestinal villus in HS_Drp1 KO group was significantly better than that in HS_WT group (Figure 2E). Moreover, the plasma D-lactic acid and endotoxin concentration in N_WT group were 0.196±0.073 mmol/L and 0.148±0.052 EU/ml, respectively, while these two indexes increased significantly in HS_WT group (p < 0.05) (D-lactic acid concentration: 0.752±0.103 mmol/L; endotoxin content: 0.595±0.131 EU/ml), further confirming the existence of intestinal barrier dysfunction after shock. After Drp1 KO, the D-lactic acid content decreased by 43% and endotoxin content decreased by 49% (p < 0.05) (Figure 2F), indicating the regulatory effect of activated Drp1 on intestinal barrier functions after shock.

**Figure 3:**
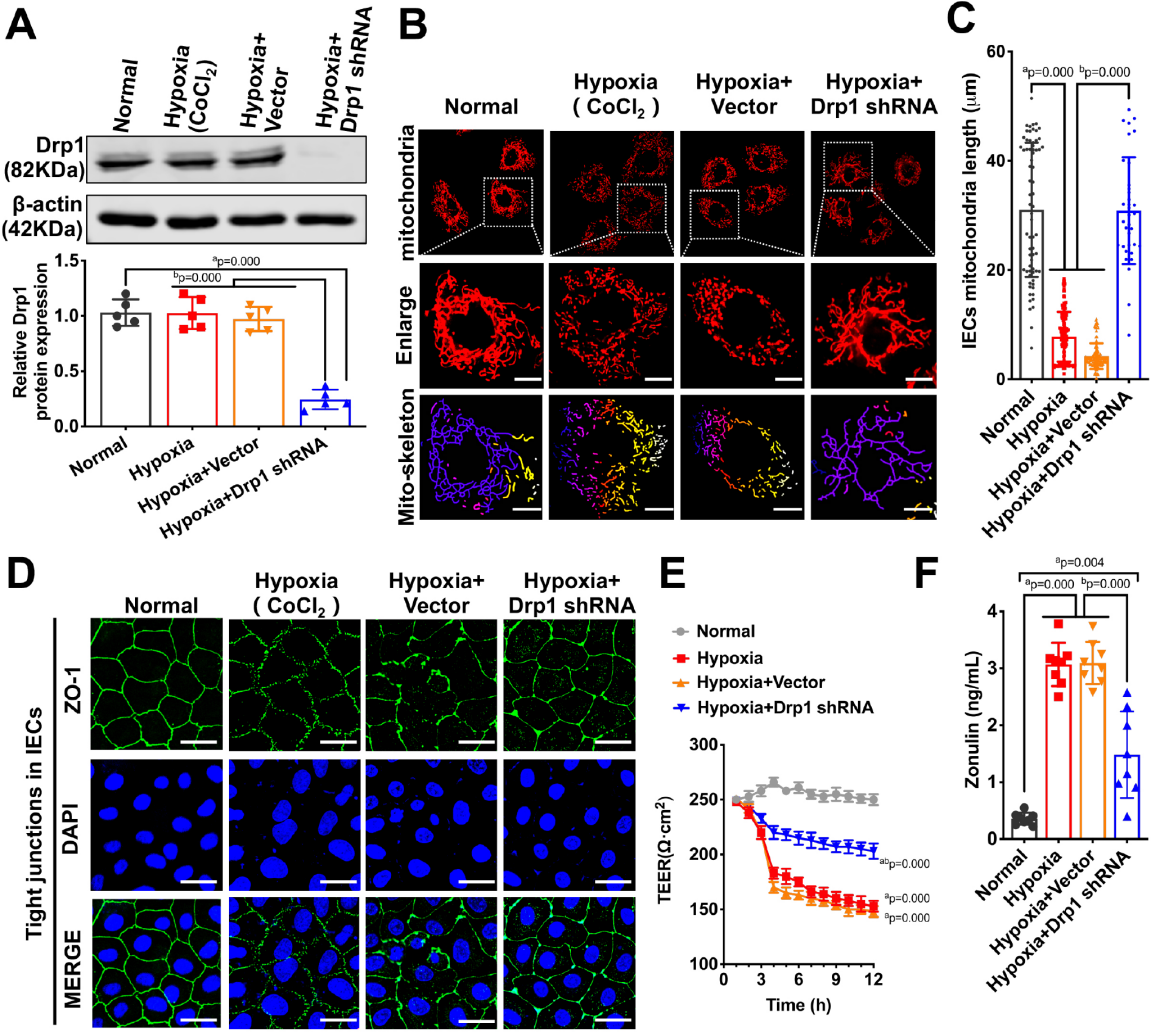
**The effects of Drp1 on intestinal epithelial tight junction after hypoxia.** (**A**) Drp1 protein expression in hypoxia-treated IECs after Drp1 shRNA (n=5). (**B**) Mitochondrial morphology in hypoxia-treated IECs after Drp1 shRNA. (Bar, 25μm). The mitochondria skeletons are analyzed by Image J software. (**C**) The length of mitochondria skeletons of IECs in each group. 69 mitochondria skeletons are observed and measured by Image J software in Normal group, 139 mitochondria in Hypoxia group, 118 mitochondria in Hypoxia+ Vector group and 35 mitochondria in Hypoxia+Drp1 shRNA group. (**D**) Tight junctions (ZO1) of IECs in each group detected by immunofluorescence. (Bar, 50μm). (**E**) The TEER value of monolayer IECs in each group. The observation time lasts 12 hours and measures every 1 hour (n=5). (**F**) The Zonulin content in supernatant of IECs in each group (n=8). a represents p < 0.05 compared with Normal group; b represents p < 0.05 compared with Hypoxia group.

To confirm the effect of activated Drp1 on intestinal epithelial tight junction after hypoxia at cellular level, we treated IECs with Drp1 shRNA and the Drp1 expression in IECs decreased by 85% (p < 0.05) (Figure 3A). The confocal images showed that mitochondrial morphology of IECs was mostly reticular in normal group and presented in short columnar in hypoxia group. After Drp1 shRNA, mitochondrial morphology was improved and reticular mitochondria increased significantly (Figure 3B). The statistical results of mitochondrial length in IECs showed that the length of mitochondria in normal group was 31.037±12.722μm and decreased to 7.778±4.338μm after hypoxia. The length of mitochondria in hypoxia+Drp1 shRNA group was improved to 30.872±9.199μm (p < 0.05) (Figure 3C). Meanwhile, it was also observed that the tight junction protein ZO1 distributed continuously along the intestinal epithelial cell membrane in normal group. In hypoxia group, the fluorescence intensity of ZO1 was weakly expressed (Supplementary Figure 2) and loosely distributed with gaps. In hypoxia+Drp1 shRNA group, the distribution of ZO1 was improved with complete structure and clear boundary (Figure 3D), suggesting the destructive effect of activated Drp1 to the tight junction in IECs after hypoxia.

We further tested the TEER value of monolayer IECs and the release of Zonulin, an intestinal permeability marker protein, to reflect the barrier function of IECs after hypoxia. The results showed that Drp1 shRNA slowed down the decline of TEER after hypoxia (Figure 3E) and Zonulin release decreased by 67% in hypoxia+Drp1 shRNA group compared with that in hypoxia group (Figure 3F). These results suggested that activated Drp1 not only affected the structure and function of mitochondria but also further regulated intestinal barrier function after hypoxia or shock.

### Activated Drp1 regulates gut microbiome composition and inhibits SCFA production after shock

**Figure 4:**
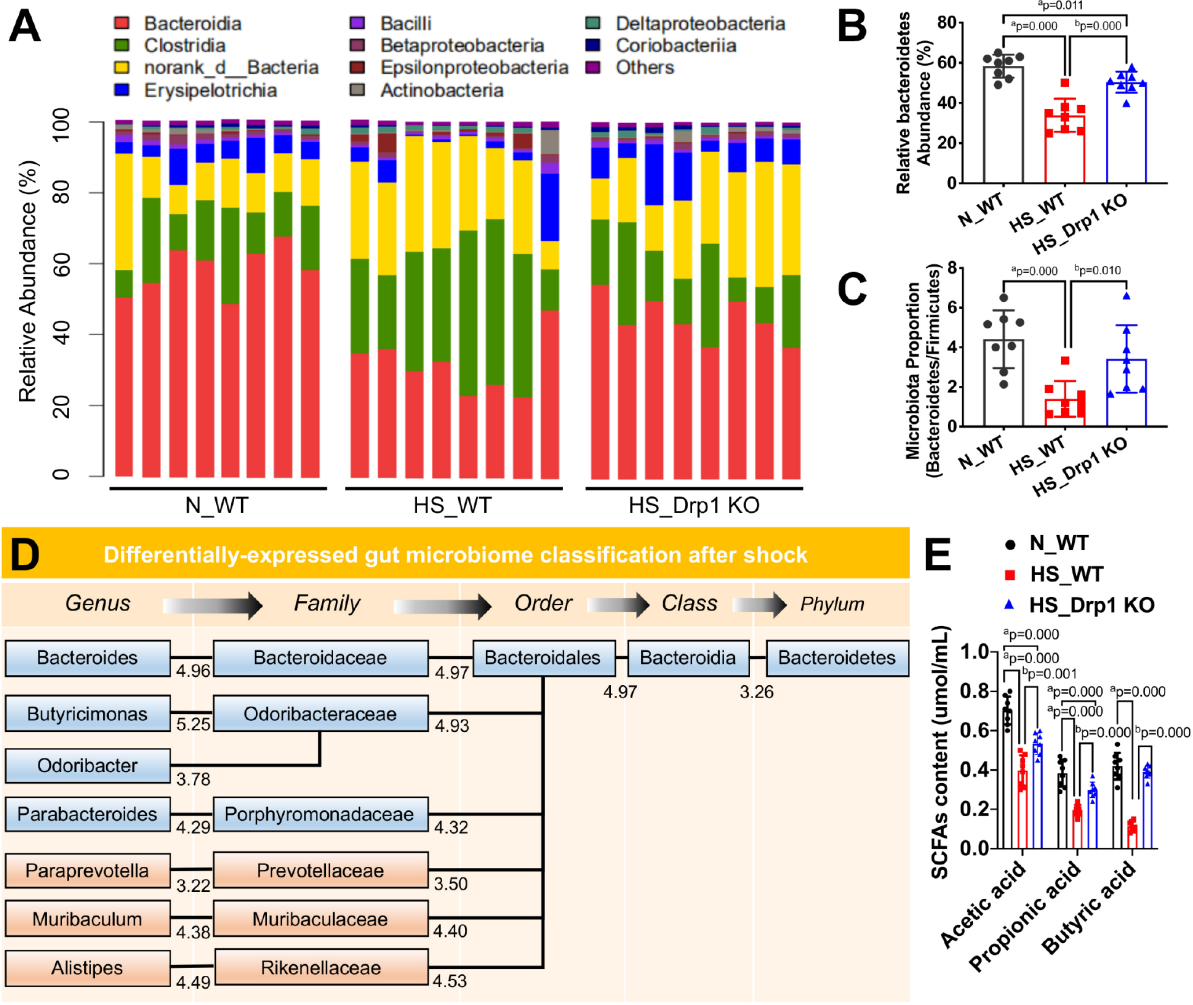
**The effects of Drp1 on gut microbiome composition and SCFA production after shock.** (**A**) The relative abundance of gut microbiome composition in each group detected by 16S rRNA gene sequencing (8 mice/ group). (**B**) The ratio of *Bacteroidetes*/*Firmicutes* in each group (8 mice/ group). (**C**) Relative *Bacteroidetes* abundance in each group detected by metagenomics profiling (8 mice/ group). (**D**) Differentially-expressed gut microbiome classification after shock detected by phylogenetic tree. The levels include phylum, class, order, family and genus. Each abundance value is labeled under branches. (**E**) The contents of intestinal SCFAs, including acetic acid, propionic acid and butyric acid, in each group (n=8 mice/ group). N_WT, WT mice in normal condition; HS_WT, WT mice in hemorrhagic shock condition; HS_Drp1 KO, Drp1 KO mice in hemorrhagic shock condition. a represents p < 0.05 compared with N_WT group; b represents p < 0.05 compared with HS_WT group.

Previous studies have shown that gut microbiome plays an important role in regulating intestinal barrier function [17–19]. To explore whether the effect of post-shock activation of Drp1 on intestinal barrier function was related to gut microbiome, we used high-throughput 16S rRNA gene sequencing and metagenomics profiling to study the differences of gut microbiome composition between N_WT group, HS_WT group and HS_Drp1 KO group (Figure 4A). The results showed that *Bacteroidetes* phylum (represented by *Bacteroidia* class, red bars in Figure 4A) was the dominant group of gut microbiome in N_WT group and *Firmicutes* phylum (represented by *Clostridia* class, green bars in Figure 4A) accounted for a certain proportion. In HS_WT group, *Bacteroidetes* still existed as dominant gut microbiome but their proportion decreased significantly. The relative abundance of *Firmicutes* increased to some extend and the *Bacteroidetes*/*Firmicutes* ratio decreased by about 68.6% (p < 0.05) (Figure 4B). Compared with HS_WT group, the abundance ratio of *Bacteroidetes*/*Firmicutes* in HS_Drp1 KO group increased by 45.2% (p < 0.05) (Figure 4B), mainly reflecting in the effect of Drp1 KO on *Bacteroidetes* phylum after shock (red bars in Figure 4A). The relative abundance of *Bacteroidetes* in N_WT group was 58.37±7.41% and decreased to 33.87±7.87% in HS_WT group. In HS_Drp1 KO group, *Bacteroidetes* abundance was markedly enhanced to 50.37±6.21% and the difference was statistically significant (p < 0.05) (Figure 4C). These results suggest that activated Drp1 in IECs may affect the composition of gut microbiome after shock.

To clarify the regulatory effect of Drp1 on gut microbiome after shock, we used phylogenetic tree to present differentially-expressed gut microbiome in *Bacteroidetes* at different levels between N_WT group and HS_WT group and each abundance value was labeled under branches (Figure 4D). Among them, the specific gut microbiome, whose abundance was up-regulated in HS_Drp1 KO group, were labeled in blue (Figure 4D). At the family level of gut microbiome, the relative abundance of *Bacteroidaceae*, *Odoribacteraceae* and *Porphyromonaceae* decreased significantly in HS_WT group and markedly increased in HS_Drp1 KO group. At the genus level, the relative abundance of *Bacteroides*, *Butyricimonas*, *Odoribacter* and *Parabacteroides* decreased in HS_WT group and improved significantly in HS_Drp1 KO group (Figure 4D). Further analysis revealed that most of the differentially-expressed gut microbiome regulated by post-shock activated Drp1 belonged to the short-chain fatty acid (SCFA) producing microbiome, which were reported to have the protective effects on tight junction and intestinal barrier function [20, 21]. To verify the above results, we tested the intestinal SCFA levels in each group. The results showed that, compared with N_WT group, the contents of acetic acid, propionic acid and butyric acid decreased by about 50% in HS_WT group (p < 0.05). In HS_Drp1 KO group, the contents of these SCFA significantly improved and the contents of butyric acid almost returned to normal level (p < 0.05) (Figure 4E). These results suggested that activated Drp1 in IECs may destroy intestinal barrier function by regulating gut microbiome composition and inhibiting the production of SCFA after shock.

### Activated Drp1 regulates gut microbiome composition and intestinal barrier function in a ROS-specific manner

**Figure 5:**
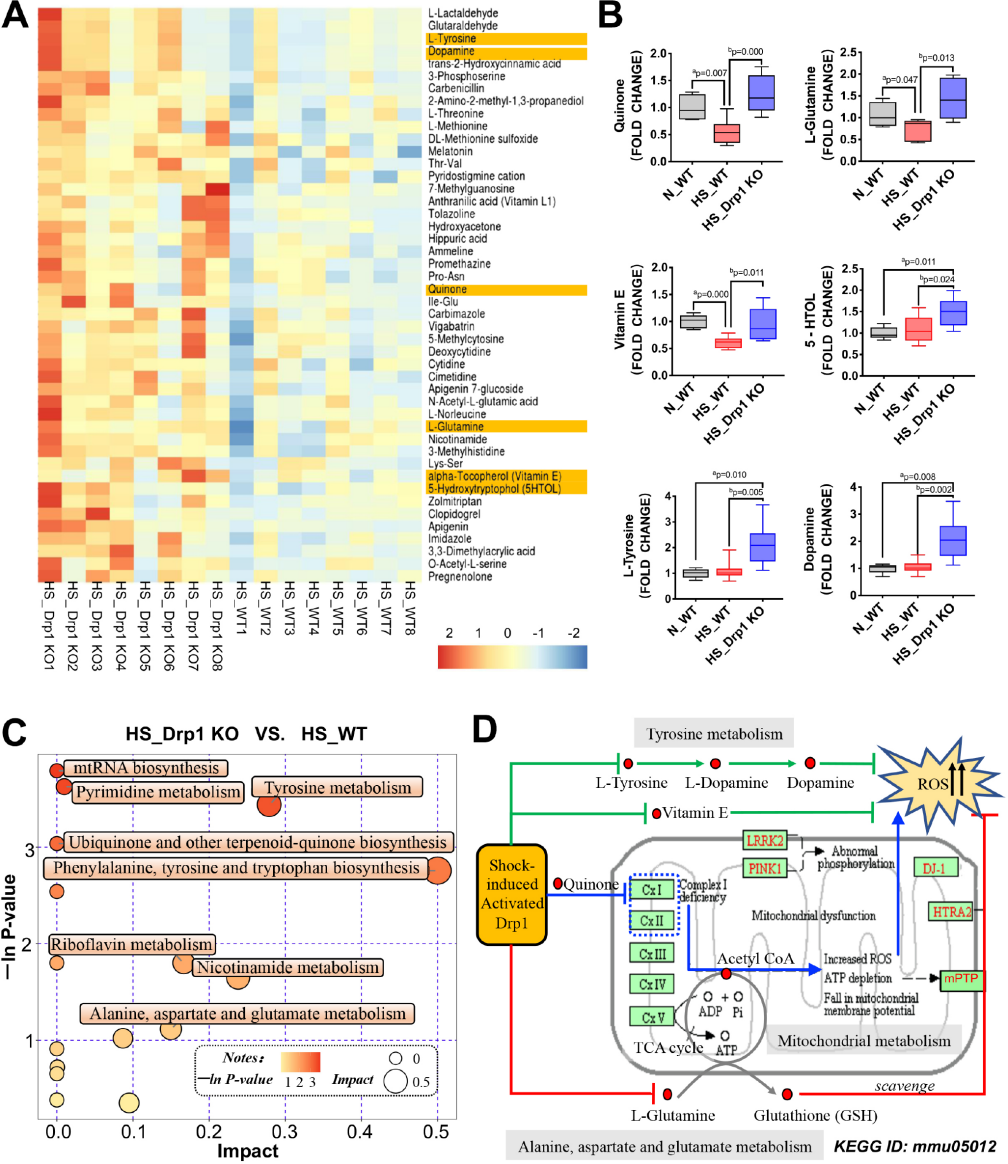
**The effects of Drp1 on mitochondrial metabolism after shock.** (**A**) Heatmap of differentially-expressed metabolites affected by Drp1 in colon tissues. Our concerned differential metabolites are labeled in yellow (8 mice/ group). (**B**) The fold change values of our concerned metabolites detected by metabolomics mass spectrometry (8 mice/ group). (**C**) The bubble map of differentially-expressed metabolic pathways affected by Drp1 in colon tissues. Each bubble represents a metabolic pathway. The abscissa and the size of bubbles indicate the impact of pathways. The bigger the bubble size is, the bigger the impact is. The ordinate and the color of bubbles indicate the *P* value of enrichment analysis (Expressed in the form of *-ln P-value*). The deeper the bubble color is, the smaller the *P* value is. (**D**) KEGG pathway annotation of differential metabolites and pathways affected by Drp1 after shock. The annotation is edited based on KEGG ID: mmu05012. N_WT, WT mice in normal condition; HS_WT, WT mice in hemorrhagic shock condition; HS_Drp1 KO, Drp1 KO mice in hemorrhagic shock condition. a represents p < 0.05 compared with N_WT group; b represents p < 0.05 compared with HS_WT group.

To explore the potential mechanisms of gut microbiome abnormality and intestinal SCFA metabolic disorder induced by activated Drp1 after shock, we used metabolomics profiling to analyze the differentially-expressed metabolites in colon tissues of HS_WT group and HS_Drp1 KO group (8 mice/group) (Figure 5A). The results showed that there were several differentially-expressed metabolites between the two groups, including Quinone, L-Glutamine, Vitamin E, L-Tyrosine, Dopamine and 5-Hydroxytryptrophol (5HTOL), etc. (Figure 5A). We further examined our concerned differential metabolites (labeled yellow) by metabolomics mass spectrometry and found that the fold change values of Quinone, L-Glutamine and Vitamin E in HS_WT group were significantly lower than those in N_WT group (p < 0.05). In HS_Drp1 KO group, the fold change values of our concerned differential metabolites were all significantly improved (p < 0.05) (Figure 5B), indicating that activated Drp1 in IECs may inhibit the expression and activity of Quinone, L-Glutamine and some other metabolites after shock.

Metabolic pathway analysis found that the most obvious differences in metabolic pathway between HS_Drp1 KO group and HS_WT group were “*Phenylalanine, tyrosine and tryptophan biosynthesis*” and “*Tyrosine metabolism*”. Other metabolic pathways including "*Ubiquinone and other terpenoid-quinone biosynthesis*", "*Alanine, aspartate and glutamate metabolism*", "*Pyrimidine metabolism*" and "*Nicotinamide metabolism*" also had some differences between these two groups to some extend (Figure 5C), suggesting that post-shock activated Drp1 in IECs may be involved in the regulation of tyrosine, ubiquinone and glutamate metabolic pathways. We also used KEGG pathway annotation to mark the above differential metabolites and found that most metabolic pathways regulated by activated Drp1 may lead to excessive accumulation of ROS (Figure 5D). The specific potential mechanisms were: (1) Activated Drp1 may disrupt mitochondrial respiratory chain by inhibiting quinone biosynthesis, leading to an increase in mitochondrial ROS production in IECs (the blue route). (2) Activated Drp1 may inhibit mitochondrial glutamate-glutathione metabolism, leading to an decrease in ROS clearance in IECs (the red route). (3) Activated Drp1 may also block some other non-mitochondrial dependent pathways, including tyrosine-dopamine metabolism and vitamin E-dependent pathway, to reduce ROS clearance in IECs (the green route) (Figure 5D). Taken together, the metabolomics profiling suggested that the potential mechanisms of Drp1-mediated gut microbiome abnormality after shock may be related to excessive ROS accumulation caused by activated Drp1 in IECs.

**Figure 6:**
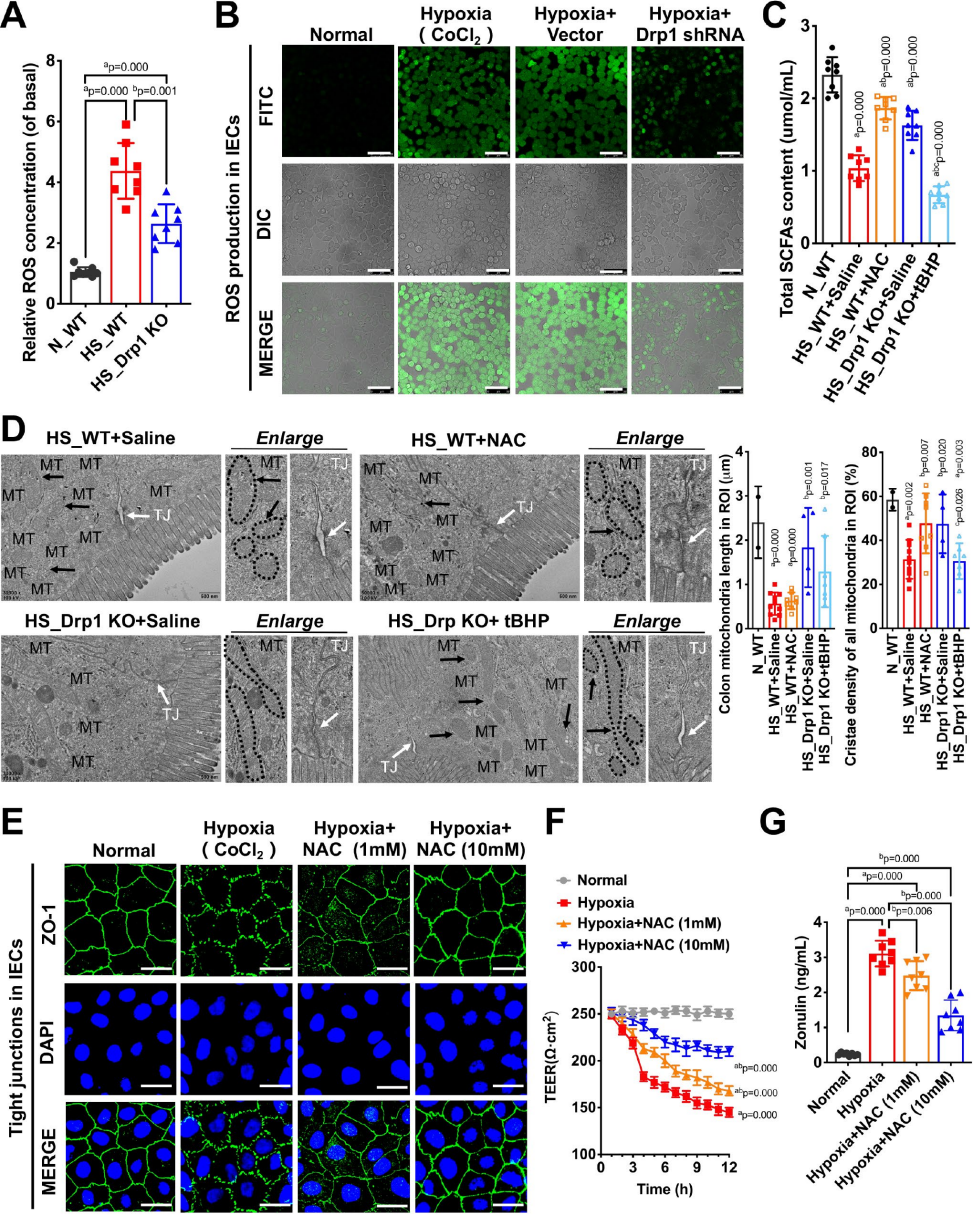
**Drp1-induced ROS accumulation is involved in the regulation of gut microbiome composition and intestinal barrier function after shock or hypoxia.** (**A**) Relative ROS concentration in colon tissues of each group (8 mice/ group). (**B**) The ROS fluorescence intensity of hypoxia-treated IECs after Drp1 shRNA (Bar, 50μm) (**C**) The content of total SCFAs after ROS intervention (8 mice/ group). (**D**) Mitochondrial morphology and tight junction of IECs after ROS intervention observed by electron microscopy. MT, mitochondria (black arrows refer to vacuolated cristae structure); TJ, tight junctions (white arrows). The boundaries of mitochondria are depicted and the lengths as well as cristae density of all mitochondria in ROI (region of interest) are calculated by Image J software. The mitochondrial number in ROI: 2 in N_WT group, 10 in HS_WT+ Saline group,8 in HS_ WT+ NAC group, 4 in HS_ Drp1 KO+ Saline group, 7 in HS_ Drp1 KO+ tBHP group. (Bar, 500nm). (**E**) Tight junctions (ZO1) of IECs after ROS intervention detected by immunofluorescence. (Bar, 50μm). (**F**) The TEER value of monolayer IECs after ROS intervention. The observation time lasts 12 hours and measures every 1 hour (n=5). (**G**) The Zonulin content in supernatant of IECs after ROS intervention (n=8). N_WT, WT mice in normal condition; HS_WT, WT mice in hemorrhagic shock condition; HS_Drp1 KO, Drp1 KO mice in hemorrhagic shock condition; NAC, N-acetylcysteine; tBHP, tert-Butyl Hydroperoxide. a represents p < 0.05 compared with N_WT or Normal group; b represents p < 0.05 compared with HS_WT, HS_WT+ Saline or Hypoxia group; c represents p < 0.05 compared with HS_Drp1 KO+ Saline group.

To verify the above metabolomics results, we detected the ROS level in colon tissues of each group and found that the ROS level in HS_WT group was 3.5 times higher than that in N_WT group while the ROS content in HS_Drp1 KO group was 40% lower than that in HS_WT group (p < 0.05) (Figure 6A). At the cellular level, the ROS fluorescence intensity of IECs increased significantly after hypoxia (p < 0.05), while the ROS level of IECs improved significantly in Hypoxia+Drp1 shRNA group (p < 0.05) (Figure 6B and Supplementary Figure 3), which was consistent with the results in tissue level. The above results indicated that Drp1 intervention may significantly reduce ROS accumulation in IECs after shock or hypoxia.

To verify whether Drp1-induced ROS accumulation was involved in the regulation of gut microbiome composition and intestinal barrier function after shock, we pre-injected *NAC* (*N-acetylcysteine*, 100mg/kg) (HS_WT+*NAC* group) or an equal volume of 0.9% saline solution (HS_WT+Saline group) intraperitoneally in WT mice before shock treatment and detected total SCFAs content after 4h shock in each group. The results showed that the content of total SCFAs in N_WT group was 2.37±0.31μmol/ml and decreased to 1.13±0.22μmol/ml in HS_WT+Saline group (p < 0.05). In HS_WT+*NAC* group, the total SCFAs contents improved by 59% (1.13±0.22μmol/ml) (p < 0.05) (Figure 6C), suggesting that removal of Drp1-induced ROS accumulation had a significant protective effect on gut microbiome and SCFAs production in shock individuals. We also pre-injected *tBHP* (*tert-Butyl Hydroperoxide*, 100μg/kg) (HS_Drp1 KO+*tBHP* group) or an equal volume of 0.9% saline solution (HS_Drp1 KO+Saline group) intraperitoneally in Drp1 KO mice before shock. The content of total SCFAs in HS_Drp1 KO+ Saline was 1.61±0.30μmol/ml and had no significant difference with that in HS_WT+*NAC* group (p > 0.05), while the SCFAs content decreased by 48% in HS_Drp1 KO+*tBHP* group (p < 0.05) (Figure 6C), demonstrating that activated Drp1-induced excessive ROS accumulation in IECs may lead to abnormal composition of gut microbiome and inhibit the production of SCFAs after shock.

To prove the regulatory effect of excessive accumulation of ROS induced by Drp1 activation on intestinal barrier function after shock, we used electron microscopy to observe mitochondrial morphology and tight junction of IECs after ROS intervention. The results showed that Removing ROS with *NAC* did not affect the length of mitochondria (black arrow) but could improve mitochondrial cristae density and tight junctions of IECs after shock (white arrow) (Figure 6D). In HS_Drp1 KO+Saline group, the length of mitochondria in IECs returned to normal level (2.71±1.09μm) and tight junction was protected by Drp1 KO from shock stimuli. However, increasing ROS with *tBHP* could disrupt the protective effect of Drp1 KO on tight junctions (white arrow) and mitochondrial cristae density but had no influence on mitochondrial length (1.83±1.05μm) (black arrow) (Figure 6D). The above results demonstrated that Drp1-induced ROS accumulation in IECs could significantly disturb intestinal barrier function after shock and this effect was not related to the morphology change of mitochondria after shock.

At the cellular level, we treated IECs with CoCl_2_ combined with *NAC* for 4 hours (Hypoxia+*NAC* group). Compared with CoCl_2_-induced hypoxia group, the fluorescence intensity of ZO-1 was markedly increased (Supplementary Figure 4) and the tight junction of IECs was significantly improved in a dose-dependent manner (Figure 6E). The TEER results indicated that scavenging ROS with NAC could significantly slow down the decline of TEER in IECs after hypoxia. Compared with Hypoxia group, TEER in Hypoxia+*NAC* (10mM) group increased by 61.9% after 12-hour observation (p < 0.05) (Figure 6F). We also tested the Zonulin content in supernatant of IECs after *NAC* and found that Zonulin release in Hypoxia+*NAC* (1mM) group and Hypoxia +*NAC* (10mM) group decreased to 82% and 44% of that in Hypoxia group, respectively (p < 0.05), (Figure 6G). The above results suggested that inhibiting Drp1-induced ROS accumulation in IECs could protect intestinal epithelial tight junction and improve intestinal barrier function after hypoxia.

## DISCUSSION

It has been widely concerned about mitochondria-microbiome interaction [8, 9, 22] and its impacts on diseases [23, 24] in recent years. Gut microbiome is the biological barrier of intestinal mucosa, which is closely related to the maintenance of intestinal homeostasis and intestinal barrier function. It is reported that mitochondria originate from symbiotic bacteria in primitive eukaryotic cells, indicating high homology between mitochondria and gut microbiome [25, 26]. Previous studies on mitochondria-microbiome interaction mainly focused on the regulation of gut microbiome metabolites on mitochondrial functions [27]. The study of Saihara K et al. found that pyrrolidine quinone synthesized by gut microbiome could stimulate mammalian mitochondrial biosynthesis through PGC1α pathway [28] and the study of Clark A et al. showed that gut microbiome could regulate mitochondrial respiration through regulating bile acid and SIRT1 expression [9]. These studies indicated that gut microbiome could regulate mitochondrial function through mitochondrial-related proteins. In this article, we focused on the feedback regulating mechanisms of mitochondrial-related protein to gut microbiome and found that Drp1-induced ROS accumulation in IECs may cause abnormal composition of *Bacteroidetes*, reduce SCFA production, and eventually lead to intestinal barrier dysfunction after shock.

There have been few reports on the feedback regulation of mitochondria to gut microbiome in the past [12, 29, 30]. Previous studies have shown that mitochondria in IECs may regulate gut microbiome via detecting bacterial infection, regulating immune response and altering genetic variation [29]. Many drugs could protect IECs from infection of gut microbiome, such as *Brucella abortus* and *Legionella pneumophila*, through regulating mitochondrial calcium signaling pathway, indicating that mitochondria are sensitive to the variation in gut microbiome [30]. The study of Ma J et al. suggested that the mutation of mitochondrial-associated proteins may essentially alter the composition and functions of gut microbiome, thereby affecting intestinal barrier function [12]. Our results on gut microbiome composition in WT mice with hemorrhagic shock showed a significant decrease in the abundance of *Bacteroidetes*. Specifically, the levels of *Bacteroides*, *Butyricimonas*, *Odoribacter*, *Parabacteroides*, *Paraprevotella*, *Muribaculum* and *Alistipes* were down-regulated and the abundance changes of the first four microbiome genus, all of which belong to SCFA-producing microbiomes [20, 21], were closely related to the activation of Drp1 after shock.

SCFAs (short-chain fatty acids) are the main products of gut microbiome, including acetic acid, propionic acid, butyric acid, isobutyric acid, valeric acid, isovaleric acid and so on [31]. Among them, acetic acid, propionic acid and butyric acid are the most abundant SCFAs in colon tissue and are reported to have protective effects on intestinal epithelial tight junction and intestinal barrier function [32]. In ulcerative colitis, a decrease in the proportion of *Bacteroidetes* down-regulated butyric acid level, thereby leading to lower expression of tight junction protein ZO1 and increased intestinal permeability [33]. In multiple sclerosis, a decrease in *Butyricimonas* content resulted in SCFAs reduction, which attenuated the anti-inflammatory effects of intestinal Treg cells [34]. The study of Eri T et al. also showed that the reduction of *Odoribacter* and *Alistipes* induced by long-term use of antibodies may damage intestinal barrier function and induce intestinal sepsis [35]. Based on these previous reports and our experimental results, we found that abnormal microbiome composition mediated by Drp1 could reduce the colon SCFAs level, resulting in the destruction of tight junction in IECs and the increase of intestinal permeability after shock. And the potential regulation mechanism was closely related to mitochondrial metabolism disorders mediated by Drp1 activation after shock.

Drp1, as an important mitochondrial-related protein, is mainly distributed in cytoplasm in the form of polymers under physiological condition [36]. Researches have shown that the expression of Drp1 was increased in multiple tumor diseases and Drp1 could regulate the migration and infiltration of tumor cells by regulating mitochondrial redistribution and the formation of plate pseudopodia [14, 37, 38]. A study in traumatic brain injury also observed that the expression of Drp1 was significantly increased but the activity of Drp1 remained unchanged [39]. However, in our study, we found that the total expression of Drp1 in IECs did not changed significantly but the activity of Drp1 increased and the translocation of Drp1 from cytoplasm to mitochondria was observed in the model of hemorrhagic shock. The difference in Drp1 expression and activity may be due to different types of disease model and tissue specificity. Previous studies have shown that there are many types of post-translational modifications in Drp1, including phosphorylation, S-nitrosylation, SUMOylation, ubiquitination and O-GlcNAcylation [16]. A study in ischemia-reperfusion injury showed that phosphorylation at Drp1 Ser616 and dephosphorylation at Drp1 Ser637 in cardiomyocytes may lead to increased ROS production and excessive mitochondrial fission [40]. In endothelial cells stimulated by hyperglycemia, phosphorylation at Drp1 Ser600 may induce mitochondrial translocation of Drp1 [41]. In our study, we detected the changes of Drp1 phosphorylation at Ser616, Ser600 and Ser637 in IECs after hemorrhagic shock. Besides, the mitochondrial translocation of Drp1 and increased ROS production were also observed in the shock model, which were consistent with previous studies in other models. In addition, our study also found that the ubiquitination of Drp1 in IECs decreased and the level of SUMOylation increased after shock. Previous studies have shown that ubiquitination of Drp1 can promote the reconstruction of mitochondrial network after cell division. When ubiquitination is inhibited, the synthesis of cytokines decreases, and the distribution of mitochondria is uneven [42]. SUMOylation of Drp1 is reported to promote the binding of Drp1 to the outer membrane of mitochondria and influence calcium exchange between mitochondria and endoplasmic reticulum [43]. Thus, the critical roles of various Drp1 modifications in the pathophysiological process of shock need to be further explored.

The production of ROS mainly occurs in mitochondrial respiratory chain. In physiological condition, excessive ROS can be scavenged by antioxidant enzymes such as superoxide dismutase, catalase, glutathione and other antioxidants, such as vitamin C and vitamin E. In pathological conditions, the increased ROS production or reduced ROS scavengement may cause excessive accumulation of ROS, resulting in oxidative stress and a series of cell dysfunction [44–46]. There have been numerous reports on Drp1-mediated ROS production [47, 48] and inhibiting Drp1 with *Mdivi* may reduce ROS level [49, 50]. Traditionally, it is believed that Drp1-induced excessive ROS production is mainly due to the disruption of mitochondrial homeostasis and excessive mitochondrial fission after Drp1 activation. But the specific regulatory mechanism is still not clear. In our study, we used metabolomics profiling and found that activated Drp1 may affect the biosynthesis of coenzyme quinone and destroy mitochondrial respiratory chain to increase ROS production after shock. Meanwhile, activated Drp1 may also reduce ROS scavengement by inhibiting mitochondrial glutamate and glutathione metabolism pathways in IECs. Our findings provide experimental evidence for how mitochondrial dynamin-related proteins specifically regulate mitochondrial functions in a metabolism way. Moreover, our study demonstrated that exogenous intervening ROS did not affect mitochondrial morphology in intestinal epithelium after shock but had great effect on SCFA level of gut microbiome and intestinal epithelial tight junctions no matter in Drp1 KO or WT mice, suggesting that the novel mechanism of ROS accumulation induced by Drp1-mediated mitochondrial metabolic pathway is relatively independent to the traditional Drp1-mediated mitochondrial morphological pathway.

In summary, our findings demonstrate that mitochondrial-associated protein Drp1 has a feedback regulatory effect on gut microbiome after hemorrhagic shock. Activated Drp1 in IECs may lead to ROS accumulation by disrupting mitochondrial respiratory chain and glutathione metabolism, which can perturb the gut microbiome composition in *Bacteroidetes* phylum. The abundance of SCFA producing microbiomes, such as *Bacteroides*, *Butyricimonas* and *Odoribacter*, decrease significantly, destroying intestinal epithelial tight junctions and intestinal barrier function after hemorrhagic shock. Our findings raise new evidence for the interaction between mitochondria and microbiome, and provide novel insights for targeting Drp1-mediated mitochondrial metabolism as well as microbiome in the treatment of intestinal barrier dysfunction after hemorrhagic shock.

## MATERIALS AND METHODS

### Animals and reagents

Thirty specific-pathogen-free C57BL/6 male mice (~ 8 weeks old, 18-25g) and thirty Drp1 knockout (Drp1 +/-) male mice (~ 8 weeks old, 20-23g) were purchased from Shanghai Model Organisms Center, Inc (Shanghai, CHINA) and housed in the animal facility of Army Medical University (Chongqing, CHINA) for a week before the start of experimentation, where they were allowed to consume pelleted rodent diet and filtered water *ad libitum* under environmental conditions of 22°C, 40–70% humidity, and a 12:12 hour light: dark cycle. The sequence of Drp1 KO identification primers (1115bp) were: 5′-GTGCCACTCGGACTGCCTTCT-3′ (Forward) and 5′-GACCTGCTCCCCACATCAACA-3’ (Reverse). All procedures were approved by the Research Council and Animal Care and Use Committee of the Research Institute of Surgery, Army Medical University (Chongqing, China).

Intestinal epithelial cell line (IECs) was obtained from the Cell Bank of the Chinese Academy of Sciences (Shanghai, CHINA) and was sub-cultured and preserved in the Army Medical Center of PLA. Antibodies for Drp1, ZO1, β-actin, ANT and ROS Detection Kit were purchased from Abcam (Cambridge, MA, USA). Antibodies for phospho-Drp1(Ser616), phospho-Drp1(Ser637), phospho-Drp1(Ser600) and Post-Translational Modification (PTM) screening kit were purchased from Cell Signaling Technology (Danvers, MA, USA). MitoTracker Deep Red, Zonulin Elisa Kit and SCFA Elisa Kit were purchased from Thermo Scientific (Waltham, MA, USA). Mitochondria Isolation Kit was purchased from Invent Biotechnologies, Inc. (Beijing, CHINA). N-acetylcysteine (NAC, a glutathione synthetic precursor to scavenge ROS [51]) and tert-Butyl Hydroperoxide (tBHP, a ROS donator [52]) were purchased from Sigma-Aldrich (St. Louis, MO, USA). Adenoviral vector for Drp1 deletion (Drp1 shRNA) was generated by Obio Technology (Shanghai, CHINA). The Drp1 shRNA sequence (5′-3′): GCTTCAAATCAGAGAACTT; the corresponding control sequence (5′-3′): TTCTCCGAACGTGTCACGT. All other chemicals were purchased from Sigma unless specifically mentioned otherwise.

### Model preparation

As for the hemorrhagic shock model preparation, twenty C57BL/6 male mice (WT) and twenty Drp1 knockout male mice (Drp1 KO) were anaesthetized with sodium pentobarbital (initial dosage, 30 mg/kg). Anaesthetized mice were placed on a warmed plate to maintain the body temperature at 37°C. Aseptic techniques were adopted for all surgical procedures. Right femoral arteries were catheterized with polyethylene catheters for bleeding 50% of total blood volume (≈7% of weight) [53]. Besides, we treated IECs with CoCl_2_ (600μM)-induced hypoxia to simulate the hemorrhagic shock state in cellular level [54].

### 16S rRNA gene sequencing

We isolated DNA from fecal pellets collected during necropsy using a PowerSoil® DNA Isolation Kit (Invitrogen, Carlsbad, CA, USA) according to the manufacturer’s instructions. The resultant DNA was quantified by ultraviolet spectroscopy and stored at -70°C for further analysis. The resulting DNA were pooled, quantified by Qubit 2.0 Fluorometer and sequenced at the Biotree Biotech Co., Ltd. (Shanghai, CHINA) using an Illumina MiSeq (500 cycles v2 kit). Paired-reads were assembled in Geneious (Biomatters, Auckland, New Zealand), followed by trimming end with error probability of 0.01 as initial quality filtering. Quantitative Insights into Microbial Ecology (QIIME) software (version 1.9.1; http://qiime.org/) was used. De novo operational taxonomic unit (OTU) picking was used and OTUs were chosen with a threshold of 97% sequence similarity. A representative sequence from each OTU was selected for taxonomic assignment according to Greengenes database (version 13_5; http://greengenes.lbl.gov/). By default, QIIME uses uclust consensus taxonomy classifier to assign taxonomy. Each representative 16S rRNA sequence was assigned by phylogenetic tree at different levels, including phylum, class, order, family and genus [55]. The macro genomics based on 16S rRNA gene sequencing was assisted by Biotree Biotech Co., Ltd. (Shanghai, CHINA).

### Metabolomics profiling

We performed LC-MS analyses on a quadrupole-time-of-flight (Q-TOF) 6510 mass spectrometer (Agilent Technologies, Santa Clara, CA) with an electrospray ionization source. The mass spectrometer was interfaced with an Agilent 1200 HPLC system. The Q-TOF was calibrated daily using the standard tuning solution from Agilent Technologies. The typical mass accuracy of the Q-TOF was < 10 ppm. Metabolites were analyzed in the positive mode only over a range of 80-1000 m/z using a C18 T3 reverse-phase column from Waters Corporation (Milford, MA). Data acquired in Agilent .d format were converted to mzXML using MassHunter Workstation software from Agilent Technologies. Data were filtered by intensity, and only signals with intensities > 1000 were considered. The converted data were processed using XCMS Online for peak picking, alignment, integration, and extraction of the peak intensities. To profile individual metabolite differences between groups, a two-tailed Welch’s t-test was used (p < 0.05). The exact masses of molecular features with significant changes were searched against Kyoto Encyclopedia of Genes and Genomes (KEGG) databases (http://www.genome.jp/kegg/) with a 10-ppm mass accuracy threshold. The matched exact masses were stored and used for the generation of MS/MS data to identify the metabolites [55, 56].

### Transmission electronic microscopy observation

Freshly colon tissues were quickly fixed with arsenate buffer containing 2.5% glutaraldehyde for 24 h (pH=7.4, 4°C). After three 10 min-wash with 0.13 M PBS, the tissues were post fixed in 1% OsO_4_ for 2 hours at room temperature and then dehydrated in a graded series of ethanol (65%, 70%, 75%, 80%, and 95% for 10 min each). After that, the tissues were incubated with tert-butoxide for 10 min and then dried with CO_2_, stained with uranyl acetate or lead citrate, coated with gold (Au) using ion sputter coater. Finally, samples were viewed and imaged with a transmission electron microscope (H-7500, Hitachi Company, Japan) [57].

### Immunofluorescence staining

Intestinal epithelial cells were plated in confocal chamber and cultured for about two days. Cells were incubated with MitoTracker Deep Red (1:10000) to stain mitochondria for 30 min at 37°C. IECs were washed twice in 1X phosphate-buffered saline (PBS) and fixed in a 4% paraformaldehyde solution for 20 min at room temperature. Cells were permeabilized with 0.1% Triton-x 100 in 1X PBS for 5 min at room temperature. Cells were then blocked in a 5% BSA solution for 1 h at room temperature, washed and incubated overnight at 4°C with primary antibodies against ZO1 and Drp1. Cells were then washed in PBS plus 0.1% Tween-20 and incubated with corresponding fluorophore-conjugated goat secondary antibodies (Invitrogen, Carlsbad, CA, USA) for 1 h at room temperature. Cells were washed as before with a final wash in 1X PBS alone and incubated with DAPI (BD Biosciences, Franklin Lakes, NJ, USA) (1:50) for 5 min at room temperature. Immunofluorescence was visualized using confocal laser-scanning microscopy (Leica SP5, Germany).

### Transepithelial electrical resistance (TEER) measurements

Intestinal epithelial cells were seeded on six-well, 3μm cell culture inserts (BD Biosciences, Franklin Lakes, NJ, USA) and TEER measurements were taken using a Millicell-ERS ohm-voltameter (Millipore, Billerica, MA, USA). The TEER value measured in non-cell chamber was regarded as blank control. IECs monolayer TEER values=(actual TEER-blank control)×Transwell area. TEER values were calculated and expressed as Ω·cm^2^.

### Mitochondrial ROS content detection

Fresh colon tissue samples of each group were washed in pre-cooled PBS. After shredding, the samples were digested with enzyme solution (Abcam, Cambridge, MA, USA) for 30 min at 37°C. After terminating the digestion with cold PBS, 300 mesh nylon mesh was used to filter out tissue mass and collect filtered cells. After centrifugation (500 G) for 10 min, the supernatant was removed and washed with PBS for 1 to 2 times. Single cell suspension was prepared by suspension and then incubated with DCFH-DA (10μM) for 30 min at 37°C. After centrifugation (1000 G) for 10 min, Cell sediments were collected fluorescence detection. As for ROS detection in IECs, cells were incubated with 1% DCFH-DA for 30 min at 37°C. After washing twice with PBS, the ROS fluorescence of IECs was visualized using confocal laser-scanning microscopy (Leica SP5, Germany).

### Statistical analysis

Principal component analysis (PCA) was performed to examine intrinsic clusters of metabolomics data. A 95% confidence interval (CI) was used as the threshold to identify potential outliers in all samples. In addition, heat maps were generated using a hierarchical clustering algorithm to visualize the metabolite difference within the data set. Data were expressed as Means and Standard Deviations (SD). One-way analysis of variance (ANOVA) was used for experiments with more than two groups and followed by Tukey’s post hoc analysis. The survival analysis was calculated by Kaplan-Meier method using SPSS 17.0 (SPSS Inc., Chicago, IL, USA). p < 0.05 was considered statistically significant.

## Supplementary Material

Supplementary Figures
