# Supplementary material for: Activated Drp1-mediated mitochondrial ROS influence the gut microbiome and intestinal barrier after hemorrhagic shock
Source: Aging (Albany NY). 2020 Jan 18;12(2):1397–416. doi: 10.18632/aging.102690 (PMC7053642; doi:10.18632/aging.102690)
Supplement: Supplementary Figures [file aging-12-102690-s001..pdf]

SUPPLEMENTARY FIGURES

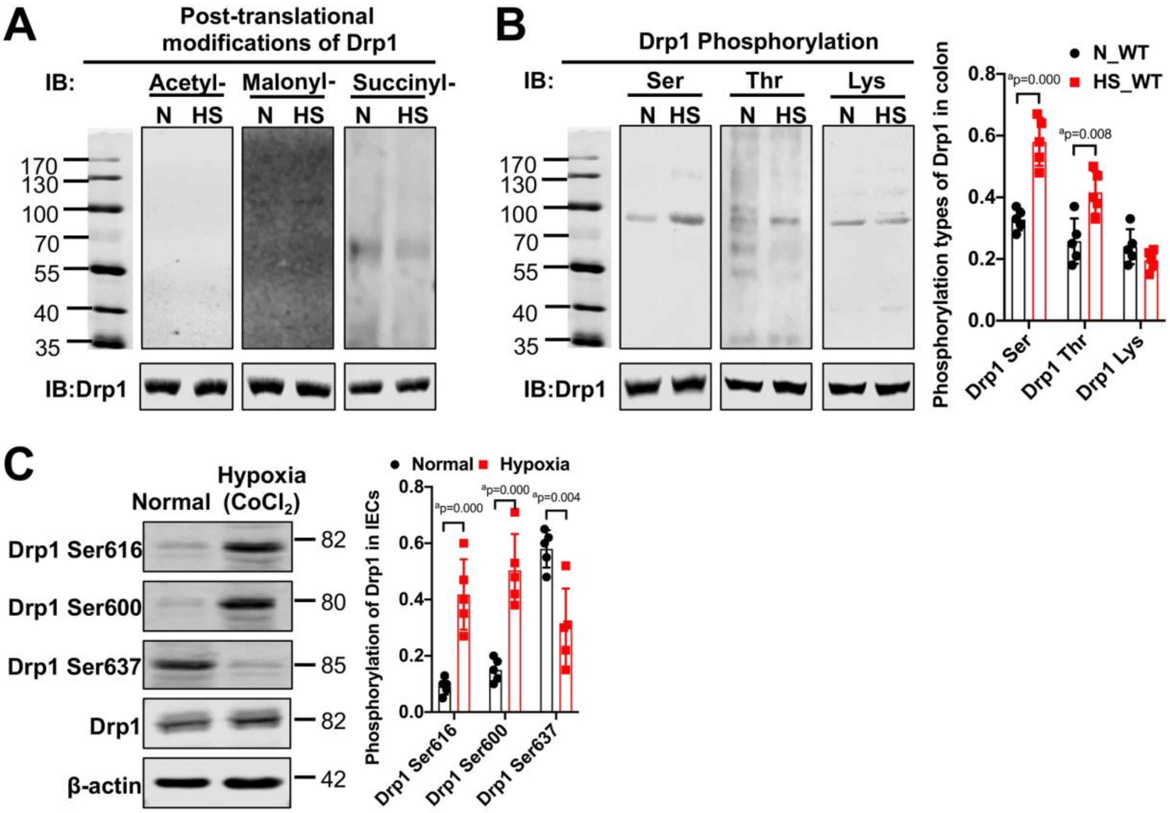

Supplementary Figure 1. Post-translational modifications of Drp1 after shock or hypoxia. (**A**) Some types of Drp1 modifications after shock in colon tissues. N, N_WT group; HS, HS_WT group; Acetyl-, acetylation; Malonyl-, malonylation; Succinyl-, succinylaction. (**B**) Different types of Drp1 phosphorylation types after shock in colon tissues. Ser, Serine; Thr, Threonine; Lys, Lysine. (n=5) (**C**) The phosphorylation of Drp1 after $CoCl_2$-induced hypoxia in IECs (n=5). a represents $p < 0.05$ compared with N_WT group or Normal group.

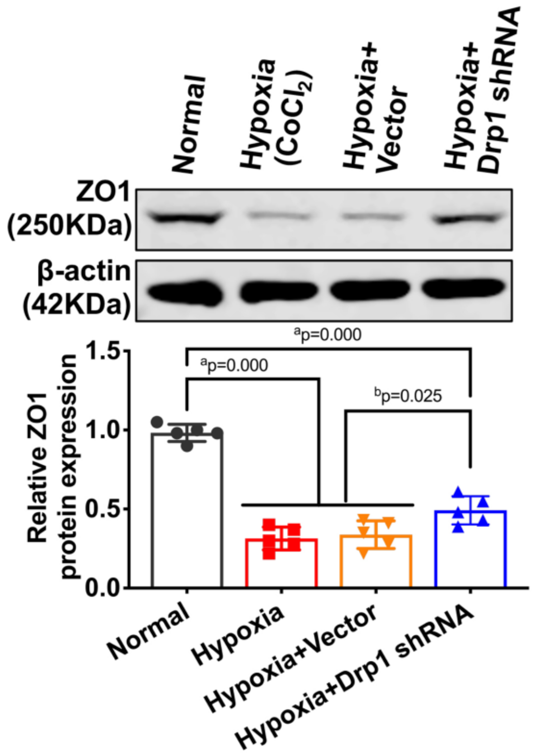

Supplementary Figure 2. The ZO1 protein expression of hypoxia-treated IECs after Drp1 shRNA. (n=5) a represents $p < 0.05$ compared with Normal group; b represents $p < 0.05$ compared with Hypoxia group.

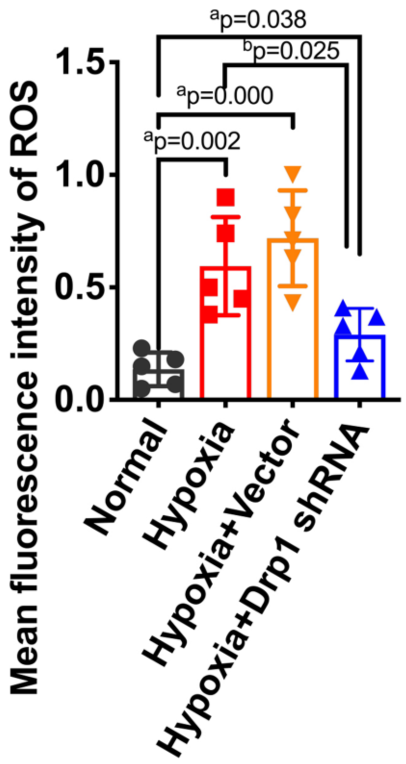

Supplementary Figure 3. The ROS fluorescence intensity of hypoxia-treated IECs after Drp1 shRNA. The statistical results of Figure 6B (n=5). a represents $p < 0.05$ compared with Normal group; b represents $p < 0.05$ compared with Hypoxia group.

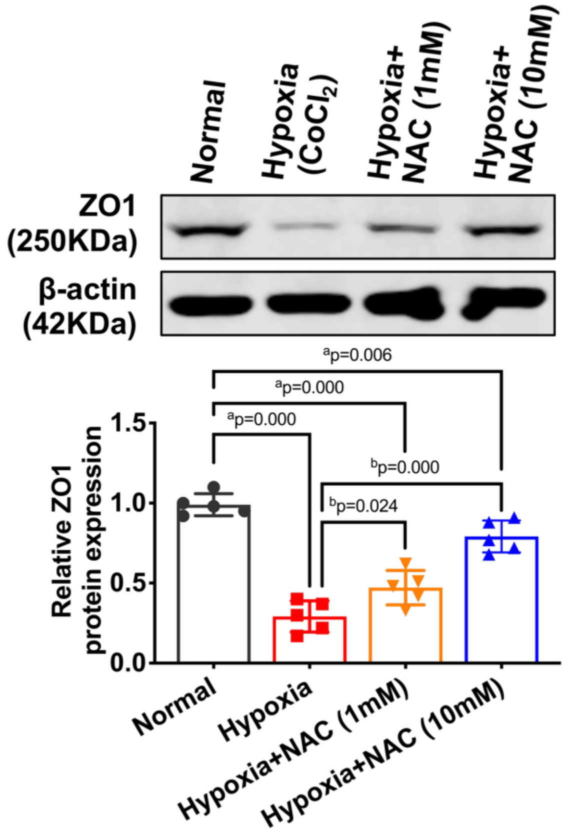

Supplementary Figure 4. The ZO1 protein expression of hypoxia-treated IECs after ROS intervention. (n=5) a represents $p < 0.05$ compared with Normal group; b represents $p < 0.05$ compared with Hypoxia group.
